# New inclusion sets for singular values

**DOI:** 10.1186/s13660-017-1337-8

**Published:** 2017-03-21

**Authors:** Jun He, Yan-Min Liu, Jun-Kang Tian, Ze-Rong Ren

**Affiliations:** School of Mathematics, Zunyi Normal College, Zunyi, Guizhou 563006 P.R. China

**Keywords:** 15A18, 15A57, 65F15, singular value, matrix, inclusion sets

## Abstract

In this paper, for a given matrix $A=(a_{ij}) \in\mathbb{C}^{n\times n}$, in terms of $r_{i}$ and $c_{i}$, where $r_{i} = \sum _{j = 1,j \ne i}^{n} {\vert {a_{ij} } \vert }$, $c_{i} = \sum _{j = 1,j \ne i}^{n} {\vert {a_{ji} } \vert }$, some new inclusion sets for singular values of the matrix are established. It is proved that the new inclusion sets are tighter than the Geršgorin-type sets (Qi in Linear Algebra Appl. 56:105-119, 1984) and the Brauer-type sets (Li in Comput. Math. Appl. 37:9-15, 1999). A numerical experiment shows the efficiency of our new results.

## Introduction

Singular values and the singular value decomposition play an important role in numerical analysis and many other applied fields [3–8]. First, we will use the following notations and definitions. Let $N :=\{1, 2, \ldots, n\}$, and assume $n \geq2$ throughout. For a given matrix $A=(a_{ij}) \in\mathbb{C}^{n\times n}$, we define $a_{i} = |a_{ii } |$, $s_{i} = \max\{r_{i}, c_{i} \} $ for any $i \in N$ and $u_{+} = \max\{0, u\}$, *u* is a real number, and where

$$r_{i} := \sum _{j = 1,j \ne i}^{n} {\vert {a_{ij} } \vert }, \qquad c_{i} := \sum _{j = 1,j \ne i}^{n} {\vert {a_{ji} } \vert }. $$

In terms of $s_{i}$, the Geršgorin-type, Brauer-type and Ky Fan-type inclusion sets of the matrix singular values are given in [1, 2, 9, 10], we list the results as follows.

### Theorem 1

*If a matrix* $A=(a_{ij}) \in\mathbb{C}^{n\times n}$, *then*

(i) (*Geršgorin*-*type*, *see* [1]) *all singular values of* *A* *are contained in*
  1 $$ C(A):=\bigcup _{i = 1}^{n} C_{i} \quad\textit{with } C_{i}=\bigl[(a_{i}-s_{i})_{+},(a_{i}+s_{i}) \bigr]\in R; $$
(ii) (*Brauer*-*type*, *see* [2]) *all singular values of* *A* *are contained in*
  2 $$ D(A):=\bigcup _{i = 1}^{n} \bigcup _{j = 1,j\neq i}^{n} \bigl\{ z\geq0: |z-a_{i}||z-a_{j}| \leq s_{i}s_{j}\bigr\} ; $$
(iii) (*Ky Fan*-*type*, *see* [2]) *let* $B=(b_{ij}) \in\mathbb{R}^{n\times n}$ *be a nonnegative matrix satisfying* $b_{ij} \geq\max\{|a_{ij}|, |a_{ji}|\}$ *for any* $i \neq j$, *then all singular values of* *A* *are contained in*
  $$E(A):=\bigcup _{i = 1}^{n} \bigl\{ z\geq0: |z-a_{i}|\leq\rho(B)-b_{ii}\bigr\} . $$

We observe that all the results in Theorem 1 are based on the values of $s_{i} = \max\{r_{i}, c_{i} \}$, if $r_{i}\ll c_{i}$ or $r_{i}\gg c_{i}$, all these singular value localization sets in Theorem 1 become very crude. In this paper, we give some new singular value localization sets which are based on the values of $r_{i}$ and $c_{i}$. The remainder of the paper is organized as follows. In Section 2, we give our main results. In Section 3, a numerical experiment is given to show the efficiency of our new results.

## New inclusion sets for singular values

Based on the idea of Li in [2], we give our main results as follows.

### Theorem 2

*If a matrix* $A=(a_{ij}) \in\mathbb{C}^{n\times n}$, *then all singular values of* *A* *are contained in*

$$\Gamma(A):=\Gamma_{1}(A) \cup\Gamma_{2}(A), $$

*where*

$$\Gamma_{1}(A):=\bigcup _{i = 1}^{n} { \bigl\{ {\sigma \ge0:\bigl\vert {\sigma^{2} - \vert {a_{ii} } \vert ^{2} } \bigr\vert \le \vert {a_{ii} } \vert r_{i} (A) + \sigma c_{i} (A)} \bigr\} } $$

*and*

$$\Gamma_{2}(A):=\bigcup _{i = 1}^{n} { \bigl\{ {\sigma \ge0:\bigl\vert {\sigma^{2} - \vert {a_{ii} } \vert ^{2} } \bigr\vert \le \vert {a_{ii} } \vert c_{i} (A) + \sigma r_{i} (A)} \bigr\} }. $$

### Proof

Let *σ* be an arbitrary singular value of *A*. Then there exist two nonzero vectors $x = (x_{1}, x_{2}, \ldots, x_{n})^{T}$ and $y = (y_{1}, y_{2},\ldots, y_{n})^{T}$ such that

3 $$ \sigma x = A^{*}y \quad\text{and} \quad\sigma y = Ax. $$

Denote

$$|x_{p}| = \max\bigl\{ |x_{i}|, 1\leq i \leq n \bigr\} ,\qquad |y_{q}| = \max\bigl\{ |y_{i}|, 1\leq i \leq n\bigr\} . $$

Now, we assume that $|x_{p}| \leq|y_{q}|$, the *q*th equations in (3) imply

4 $$\begin{aligned}& \sigma x_{q}-\overline{a}_{qq}y_{q}= \sum _{j = 1,j \ne q}^{n} {\overline {a}_{jq} } y_{j}, \end{aligned}$$

5 $$\begin{aligned}& \sigma y_{q}-a_{qq}x_{q}=\sum _{j = 1,j \ne q}^{n} {a_{qj} } x_{j}. \end{aligned}$$

Solving for $y_{q}$ we can get

6 $$ \bigl( \sigma^{2}-a_{qq}\overline{a}_{qq} \bigr)y_{q}=a_{qq}\sum _{j = 1,j \ne q}^{n} {\overline{a}_{jq} } y_{j} +\sigma\sum _{j = 1,j \ne q}^{n} {a_{qj} } x_{j}. $$

Taking the absolute value on both sides of the equation and using the triangle inequality yield

7 $$ \bigl\vert \sigma^{2}-|a_{qq}|^{2} \bigr\vert |y_{q}| \leq|a_{qq}|\sum _{j = 1,j \ne q}^{n} {|\overline{a}_{jq}| } |y_{j} |+\sigma\sum _{j = 1,j \ne q}^{n} {|a_{qj}| } |x_{j}|. $$

Then we can get

$$\bigl\vert \sigma^{2}-|a_{qq}|^{2} \bigr\vert \leq|a_{qq}|c_{q} (A) + \sigma r_{q} (A). $$

Similarly, if $|y_{q}|\leq|x_{p}|$, we can get

$$\bigl\vert \sigma^{2}-|a_{pp}|^{2} \bigr\vert \leq \vert {a_{pp} } \vert r_{p} (A) + \sigma c_{p} (A). $$

Thus, we complete the proof. □

### Remark 1

Since

$$\vert {a_{ii} } \vert r_{i} (A) + \sigma c_{i} (A) \leq \bigl(\vert {a_{ii} } \vert + \sigma \bigr)s_{i} $$

and

$$\vert {a_{ii} } \vert c_{i} (A) + \sigma r_{i} (A) \leq \bigl(\vert {a_{ii} } \vert + \sigma \bigr)s_{i}, $$

the results in Theorem 2 are always better than the results in Theorem 1(i).

### Theorem 3

*If a matrix* $A=(a_{ij}) \in\mathbb{C}^{n\times n}$, *then all singular values of* *A* *are contained in*

$$\Omega(A):=\Omega_{1}(A) \cup\Omega_{2}(A) \cup\Omega_{3}(A), $$

*where*

$$\begin{aligned}& \begin{aligned} \Omega_{1}(A):={}&\bigcup _{i \neq j} \bigl\{ \sigma \ge0:\\ &\bigl\vert {\sigma^{2} - \vert {a_{ii} } \vert ^{2} }\bigr\vert \bigl\vert {\sigma^{2} - \vert {a_{jj} } \vert ^{2} }\bigr\vert \le \bigl(\vert {a_{ii} } \vert r_{i} (A) + \sigma c_{i} (A) \bigr) \bigl(\vert {a_{jj} } \vert r_{j} (A) + \sigma c_{j} (A) \bigr) \bigr\} , \end{aligned}\\& \begin{aligned} \Omega_{2}(A):={}&\bigcup _{i \neq j} \bigl\{ \sigma \ge0:\\ &\bigl\vert {\sigma^{2} - \vert {a_{ii} } \vert ^{2} }\bigr\vert \bigl\vert {\sigma^{2} - \vert {a_{jj} } \vert ^{2} }\bigr\vert \le \bigl( \vert {a_{ii} } \vert c_{i} (A) + \sigma r_{i} (A) \bigr) \bigl( \vert {a_{jj} } \vert c_{j} (A) + \sigma r_{j} (A) \bigr) \bigr\} , \end{aligned}\\& \begin{aligned} \Omega_{3}(A):={}&\bigcup _{i \neq j} \bigl\{ \sigma \ge0:\\ &\bigl\vert {\sigma^{2} - \vert {a_{ii} } \vert ^{2} }\bigr\vert \bigl\vert {\sigma^{2} - \vert {a_{jj} } \vert ^{2} }\bigr\vert \le \bigl(\vert {a_{ii} } \vert c_{i} (A) + \sigma r_{i} (A) \bigr) \bigl( \vert {a_{jj} \vert c_{j} (A) + \sigma r_{j} (A) \bigr) } \bigr\} \end{aligned} \end{aligned}$$

*and*

$$\begin{aligned} \Omega_{4}(A):={}&\bigcup _{i \neq j} \bigl\{ \sigma \ge0:\\ &\bigl\vert {\sigma^{2} - \vert {a_{ii} } \vert ^{2} }\bigr\vert \bigl\vert {\sigma^{2} - \vert {a_{jj} } \vert ^{2} }\bigr\vert \le \bigl(\vert {a_{ii} } \vert r_{i} (A) + \sigma c_{i} (A) \bigr) \bigl( \vert {a_{jj} } \vert c_{j} (A) + \sigma r_{j} (A) \bigr) \bigr\} . \end{aligned}$$

### Proof

Let *σ* be an arbitrary singular value of *A*. Then there exist two nonzero vectors $x = (x_{1}, x_{2}, \ldots, x_{n})^{T}$ and $y = (y_{1}, y_{2},\ldots, y_{n})^{T}$ such that

8 $$ \sigma x = A^{*}y \quad\text{and} \quad\sigma y = Ax. $$

Denote $\omega_{i} = \max\{|x_{i}|, |y_{i}|\}$. Let *q* be an index such that $\omega_{q}= \max\{|\omega_{i}|, i \in N\}$. Obviously, $\omega_{q}\neq0$. Let *p* be an index such that $\omega_{p} = \max\{|\omega_{i}|, i \in N, i\neq q\}$.

Case I: We suppose $\omega_{q}=|x_{q}|$, $\omega_{p}=|x_{p}|$, similar to the proof of Theorem 2, the *q*th equations in (8) imply

9 $$\begin{aligned} \bigl\vert \sigma^{2}-|a_{qq}|^{2} \bigr\vert \omega_{q} &\leq|a_{qq}|\sum _{j = 1,j \ne q}^{n} {|a_{qj}| } |y_{j} |+\sigma \sum _{j = 1,j \ne q}^{n} {|a_{jq}| } |x_{j}| \\ &\leq \Biggl( |a_{qq}|\sum _{j = 1,j \ne q}^{n} {|a_{qj}| } +\sigma \sum _{j = 1,j \ne q}^{n} {|a_{jq}| } \Biggr) \omega_{p}. \end{aligned}$$

Similarly, the *p*th equations in (8) imply

10 $$ \bigl\vert \sigma^{2}-|a_{pp}|^{2} \bigr\vert \omega_{p} \leq \Biggl( |a_{pp}|\sum _{j = 1,j \ne p}^{n} {|a_{pj}| } +\sigma\sum _{j = 1,j \ne p}^{n} {|a_{jp}| } \Biggr) \omega_{q}. $$

Multiplying inequalities (9) with (10), we have

$$\bigl\vert {\sigma^{2} - \vert {a_{pp} } \vert ^{2} }\bigr\vert \bigl\vert {\sigma ^{2} - \vert {a_{qq} } \vert ^{2} }\bigr\vert \le \bigl(\vert {a_{pp} } \vert r_{p} (A) + \sigma c_{p} (A) \bigr) \bigl( \vert {a_{qq} } \vert r_{q} (A) + \sigma c_{q} (A) \bigr). $$

Case II: We suppose $\omega_{q}=|y_{q}|$, $\omega_{p}=|y_{p}|$, similar to the proof of Theorem 2, the *q*th equations in (8) imply

11 $$\begin{aligned} \bigl\vert \sigma^{2}-|a_{qq}|^{2} \bigr\vert \omega_{q} &\leq|a_{qq}|\sum _{j = 1,j \ne q}^{n} {|a_{jq}| } |y_{j} |+\sigma \sum _{j = 1,j \ne q}^{n} {|a_{qj}| } |x_{j}| \\ &\leq \Biggl( |a_{qq}|\sum _{j = 1,j \ne q}^{n} {|a_{jq}| } +\sigma \sum _{j = 1,j \ne q}^{n} {|a_{qj}| } \Biggr) \omega_{p}. \end{aligned}$$

Similarly, the *p*th equations in (8) imply

12 $$ \bigl\vert \sigma^{2}-|a_{pp}|^{2} \bigr\vert \omega_{p} \leq \Biggl( |a_{pp}|\sum _{j = 1,j \ne p}^{n} {|a_{jp}| } +\sigma\sum _{j = 1,j \ne p}^{n} {|a_{pj}| } \Biggr) \omega_{q}. $$

Multiplying inequalities (11) with (12), we have

$$\bigl\vert {\sigma^{2} - \vert {a_{pp} } \vert ^{2} }\bigr\vert \bigl\vert {\sigma ^{2} - \vert {a_{qq} } \vert ^{2} }\bigr\vert \le \bigl( \vert {a_{pp} } \vert c_{p} (A) + \sigma r_{p} (A) \bigr) \bigl( \vert {a_{qq} } \vert c_{q} (A) + \sigma r_{q} (A) \bigr). $$

Case III: We suppose $\omega_{q}=|y_{q}|$, $\omega_{p}=|x_{p}|$, similar to the proof of Theorem 2, the *q*th equations in (8) imply

13 $$\begin{aligned} \bigl\vert \sigma^{2}-|a_{qq}|^{2} \bigr\vert \omega_{q} &\leq|a_{qq}|\sum _{j = 1,j \ne q}^{n} {|a_{jq}| } |y_{j} |+\sigma \sum _{j = 1,j \ne q}^{n} {|a_{qj}| } |x_{j}| \\ &\leq \Biggl( |a_{qq}|\sum _{j = 1,j \ne q}^{n} {|a_{jq}| } +\sigma \sum _{j = 1,j \ne q}^{n} {|a_{qj}| } \Biggr) \omega_{p}. \end{aligned}$$

Similarly, the *p*th equations in (8) imply

14 $$ \bigl\vert \sigma^{2}-|a_{pp}|^{2} \bigr\vert \omega_{p} \leq \Biggl( |a_{pp}|\sum _{j = 1,j \ne p}^{n} {|a_{pj}| } +\sigma\sum _{j = 1,j \ne p}^{n} {|a_{jp}| } \Biggr) \omega_{q}. $$

Multiplying inequalities (13) with (14), we have

$$\bigl\vert {\sigma^{2} - \vert {a_{pp} } \vert ^{2} }\bigr\vert \bigl\vert {\sigma ^{2} - \vert {a_{qq} } \vert ^{2} }\bigr\vert \le \bigl(\vert {a_{pp} } \vert r_{p} (A) + \sigma c_{p} (A) \bigr) \bigl( \vert {a_{qq} } \vert c_{q} (A) + \sigma r_{q} (A) \bigr). $$

Case IV: We suppose $\omega_{q}=|x_{q}|$, $\omega_{p}=|y_{p}|$, similar to the proof of Cases I, II, III, we can get

$$\bigl\vert {\sigma^{2} - \vert {a_{pp} } \vert ^{2} }\bigr\vert \bigl\vert {\sigma ^{2} - \vert {a_{qq} } \vert ^{2} }\bigr\vert \le \bigl(\vert {a_{pp} } \vert c_{p} (A) + \sigma r_{p} (A) \bigr) \bigl( \vert {a_{qq} } \vert c_{q} (A) + \sigma r_{q} (A) \bigr). $$

Thus, we complete the proof. □

### Remark 2

Since

$$\begin{aligned}& \bigl(\vert {a_{ii} } \vert r_{i} (A) + \sigma c_{i} (A) \bigr) \bigl(\vert {a_{jj} } \vert r_{j} (A) + \sigma c_{j} (A) \bigr) \leq \bigl(\vert {a_{ii} } \vert + \sigma \bigr) \bigl(\vert {a_{jj} } \vert + \sigma \bigr)s_{i}s_{j}, \\& \bigl( \vert {a_{ii} } \vert c_{i} (A) + \sigma r_{i} (A) \bigr) \bigl( \vert {a_{jj} } \vert c_{j} (A) + \sigma r_{j} (A) \bigr) \leq \bigl(\vert {a_{ii} } \vert + \sigma \bigr) \bigl(\vert {a_{jj} } \vert + \sigma \bigr)s_{i}s_{j}, \\& \bigl(\vert {a_{ii} } \vert r_{i} (A) + \sigma c_{i} (A) \bigr) \bigl( \vert {a_{jj} } \vert c_{j} (A) + \sigma r_{j} (A) \bigr) \leq \bigl(\vert {a_{ii} } \vert + \sigma \bigr) \bigl(\vert {a_{jj} } \vert + \sigma \bigr)s_{i}s_{j} \end{aligned}$$

and

$$\bigl(\vert {a_{ii} } \vert r_{i} (A) + \sigma c_{i} (A) \bigr) \bigl( \vert {a_{jj} } \vert c_{j} (A) + \sigma r_{j} (A) \bigr) \leq \bigl(\vert {a_{ii} } \vert + \sigma \bigr) \bigl(\vert {a_{jj} } \vert + \sigma \bigr)s_{i}s_{j}, $$

the results in Theorem 3 are always better than the results in Theorem 1(ii).

We now establish comparison results between $\Gamma(A)$ and $\Omega(A)$.

### Theorem 4

*If a matrix* $A=(a_{ij}) \in\mathbb{C}^{n\times n}$, *then*

$$\sigma(A) \in \Omega(A) \subseteq\Gamma(A). $$

### Proof

Let *z* be any point of $\Omega_{3} (A)$. Then there are $i , j \in N$, $i \neq j$, such that $z\in\Omega_{3} (A)$, i.e.,

15 $$ \bigl\vert {z ^{2} - \vert {a_{ii} } \vert ^{2} }\bigr\vert \bigl\vert {z ^{2} - \vert {a_{jj} } \vert ^{2} }\bigr\vert \le \bigl(\vert {a_{ii} } \vert r_{i} (A) + z c_{i} (A) \bigr) \bigl( \vert {a_{jj} } \vert c_{j} (A) + z r_{j} (A) \bigr). $$

If $(\vert {a_{ii} } \vert r_{i} (A) + z c_{i} (A) ) ( \vert {a_{jj} } \vert c_{j} (A) + z r_{j} (A) )=0$, then

$$\bigl\vert {z ^{2} - \vert {a_{ii} } \vert ^{2} }\bigr\vert =0 $$

or

$$\bigl\vert {z ^{2} - \vert {a_{jj} } \vert ^{2} }\bigr\vert =0. $$

Therefore, $z \in\Gamma_{1} (A)\cup\Gamma_{2} (A)$. Moreover, if $(\vert {a_{ii} } \vert r_{i} (A) + z c_{i} (A) ) ( \vert {a_{jj} } \vert c_{j} (A) + z r_{j} (A) )> 0$, then from inequality (15), we have

16 $$ \frac{{\vert {z^{2} - \vert {a_{ii}^{2} } \vert } \vert }}{{\vert {a_{ii} } \vert r_{i} (A) + z c_{i} (A)}}\frac{{\vert {z^{2} - \vert {a_{jj}^{2} } \vert } \vert }}{{\vert {a_{jj} } \vert c_{j} (A) + z r_{j} (A)}} \le1. $$

Hence, from inequality (16), we have that

$$\frac{{\vert {z^{2} - \vert {a_{ii}^{2} } \vert } \vert }}{{\vert {a_{ii} } \vert r_{i} (A) + z c_{i} (A)}} \le1 $$

or

$$\frac{{\vert {z^{2} - \vert {a_{jj}^{2} } \vert } \vert }}{{\vert {a_{jj} } \vert c_{j} (A) + z r_{j} (A)}} \le1. $$

That is, $z \in\Gamma_{1} (A)$ or $z \in\Gamma_{2} (A)$, i.e., $z \in \Gamma(A)$.

Similarly, if *z* is any point of $\Omega_{1} (A)$ or $\Omega_{2} (A)$, we can get

$$\sigma(A) \in \Omega_{1}(A) \subseteq\Gamma(A) $$

and

$$\sigma(A) \in \Omega_{2}(A) \subseteq\Gamma(A). $$

Thus, we complete the proof. □

## Numerical example

### Example 1

Let

$$A=\left [ { \textstyle\begin{array}{c@{\quad}c} 1 & 4 \\ {0.1} & {0.5} \end{array}\displaystyle } \right ]. $$

**Figure 1:**
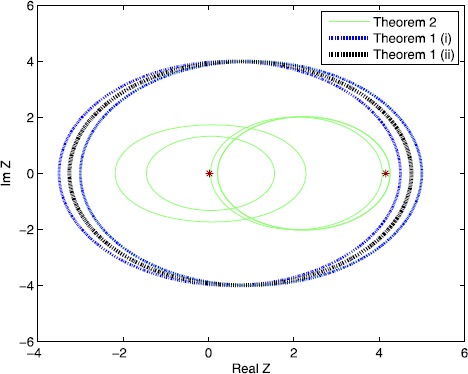
**Comparisons of Theorem** **1** **(i), Theorem** **1** **(ii) and Theorem** **2** **for Example** **1** **.**

**Figure 2:**
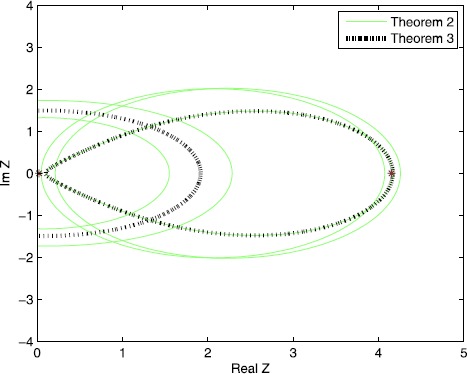
**Comparisons of Theorem** **2** **and Theorem** **3** **(** $\pmb{\Omega_{3}}$ **) for Example** **1** **.**

The singular values of *A* are $\sigma_{1} = 4.1544$ and $\sigma_{2} = 0.0241$. From Figure 1, it is easy to see that Theorem 2 is better than Theorem 1 for certain examples. In Figure 2, we can see that the results in Theorem 3 are tighter than the results in Theorem 2, which is analyzed in Theorem 4.

## Conclusion

In this paper, some new inclusion sets for singular values are given. Theoretical analysis and numerical example show that these estimates are more efficient than recent corresponding results in some cases.
